# Bioinformatic Analyses and Experimental Verification Reveal that High FSTL3 Expression Promotes EMT *via* Fibronectin-1/α5β1 Interaction in Colorectal Cancer

**DOI:** 10.3389/fmolb.2021.762924

**Published:** 2021-11-24

**Authors:** Yuanjie Liu, Jiepin Li, Shuhong Zeng, Ying Zhang, Yonghua Zhang, Zhichao Jin, Shenlin Liu, Xi Zou

**Affiliations:** ^1^ Affiliated Hospital of Nanjing University of Chinese Medicine, Jiangsu Province Hospital of Chinese Medicine, Nanjing, China; ^2^ No. 1 Clinical Medical College, Nanjing University of Chinese Medicine, Nanjing, China; ^3^ Department of Oncology, Zhangjiagang TCM Hospital Affiliated to Nanjing University of Chinese Medicine, Zhangjiagang, China

**Keywords:** colorecal cancer, FSTL3 gene, EMT—epithelial to mesenchymal transformation, FN1, fibronectin 1, α5β1 integrin, actin, M2 macrophage, prognosis (carcinoma)

## Abstract

**Background:** Colorectal cancer (CRC) is a typical cancer prevalent worldwide. Despite the conventional treatments, CRC has a poor prognosis due to relapse and metastasis. Moreover, there is a dearth of sensitive biomarkers for predicting prognosis in CRC.

**Methods:** This study used a bioinformatics approach combining validation experiments to examine the value of follistatin-like 3 (*FSTL3*) as a prognostic predictor and therapeutic target in CRC.

**Results:** *F*STL3 was remarkably upregulated in the CRC samples. FSTL3 overexpression was significantly associated with a poor prognosis. FSTL3 was found to activate the epithelial-mesenchymal transition by promoting the binding of FN1 to α5β1. *FSTL3* expression was also positively correlated with the abundance of the potent immunosuppressors, M2 macrophages.

**Conclusion:** *FSTL3* overexpression affects CRC prognosis and thus, *FSTL3* can be a prognostic biomarker and therapeutic target with potential applications in CRC.

## 1 Introduction

Colorectal cancer (CRC) is the third most common cancer globally (Bray et al., 2020; Chen et al., 2020). The CRC patients undergo cancer-related mortality due to frequent metastasis in the liver, lungs, and further distant regions (Brody, 2015). The predisposing factors for CRC like smoking, unhealthy diets, obesity epidemic, and lack of exercise, are now rampant in the high-income industrialized countries (Wieszczy et al., 2017). Other risk factors, such as genetic mutations colorectal adenomatous polyposis (APC), deletion of K-RAS, p53, protooncogene serine/threonine kinase (BRAF), mismatch repair (MMR) gene, and microsatellite instability (MSI), triggering colon cancer (Harada and Morlote, 2020). The effective early diagnosis of CRC is limited, despite the conventional colonoscopy screening (The Lancet Gastroenterology and Hepatology, 2017). The early symptoms and signs of colon cancer are not distinct with most CRC patients being diagnosed in their middle to late stages. The conventional treatments for CRC include surgery, radiotherapy, and drug therapy. The latter includes chemical drugs, small molecule drugs targeting mutant genes, small molecule drugs targeting signal pathways, drugs targeting epigenetic regulation, and immune checkpoint inhibitors (Piawah and Venook, 2019). Recent studies based on the molecularly targeted drugs Bevacizumab, Cetuximab and Sorafenib have been used for treating CRC. These drugs have improved the overall survival rate of CRC patients (Garcia et al., 2020; Jeong et al., 2020; Giordano et al., 2021). However, not all patients, especially the ones with advanced and distant metastases, benefit from these targeted drugs. This necessitates the exploration of the new biomarkers or therapeutic targets, for improving the personalized systemic treatments.

*FSTL3*, also known as *FLRG*, is a protein-coding gene located in the chromosome q 13.3 region (Maguer-Satta and Rimokh, 2004). *FSTL3* has been confirmed as an oncogene closely associated with the proliferation and metastasis of the tumor cell (Bloise et al., 2009; Gao et al., 2020). The rapid development of bioinformatics in recent years along with the increasing availability of transcriptomic data and clinical information have developed favorable conditions for investigating cancer pathogenesis. Our team has been involved in exploring the digestive system cancer pathogenesis (Bloise et al., 2009; Li et al., 2016). Our team previously found that FSTL3 is highly expressed in gastric cancer, promoting epithelial-mesenchymal transition (EMT) through the BMP/SMAD signaling pathway. In addition, the FSTL3 overexpression is known to promote M2 macrophage infiltration in the tumor microenvironment (Liu et al., 2021). Considering the role of FSTL3 in gastric cancer, we hypothesized that FSTL3 may have a similar molecular mechanism in CRC, which was explored in this study. We found that the expression level of FSTL3 was significantly elevated in CRC and was an independent prognostic factor for CRC patients. More importantly, FSTL3 was identified as a key factor in the remodeling of the CRC tumor microenvironment and a promising therapeutic target for blocking CRC metastasis.

## 2 Materials and Methods

### 2.1 The Research Flowchart

Supplementary Figure S1 shows the workflow of our study and Supplementary Figure S2 illustrates the mechanism of our study.

### 2.2 Transcriptomic Expression Analysis

The FSTL3 expression in CRC was first investigated using the TIMER web tool (https://cistrome.shinyapps.io/timer/) (Li et al., 2017) and the Gene Expression Profiling Interactive Analysis (GEPIA) database (https://www.oncomine.org/) (Tang et al., 2017). The differential expression of FSTL3 was further confirmed using the TCGA-Colon Adenocarcinoma (COAD) cohort and GSE10950, GSE44861 datasets (Zhao et al., 2020; Chen and Ke, 2021).

### 2.3 Cox Model Establishment and Prognostic Significance Analysis

The raw counts of RNA-sequencing data and corresponding clinical information regarding *FSTL3* were obtained from the TCGA dataset (https://portal.gdc.cancer.gov/) in January 2020 (Wang et al., 2016). The univariate and multivariate cox regression analyses were performed to access the independent prognostic values of *FSTL3*. The forest plot represented the *p*-value, hazard ratio (HRs), and 95% confidence interval (CIs) of each variable through the “forestplot” R package.

The *FSTL3* expression levels were analyzed based on the various classification parameters, such as the T (Tumor) stages, N (Node) stages, M (Metastasis) stages, pathological stages, and histological grades according to the TCGA-COAD data.

To reveal the value of *FSTL3* on the prognosis of CRC patients, survival analyses such as the overall survival (OS), disease-free survival (DFS), and post-progression survival (PPS) were performed through GEPIA. The automatically selected best cutoff was selected for analysis. In addition, the progression-free survival (PFS), progression-free interval (PFI), disease-specific survival (DSS), and disease-free interval (DFI) were also estimated based on the colon cancer cases in TCGA-COAD.

### 2.4 FSTL3-Correlated Gene Enrichment Analysis

The median cutoff of *FSTL3* expression in the TCGA-COAD was used to define the groups with high and low *FSTL3* expression. The “DESeq” R package was used to obtain the *FSTL3* -correlated genes. The “Enrichr” database was used to perform the functional enrichment analysis to explore the potential functions of *FSTL3*(Kuleshov et al., 2016).

The gene set enrichment analysis (GSEA) was performed using the Broad Institute GSEA software 3.0 (Powers et al., 2018). The gene set “subset of GO” was downloaded from the Molecular Signatures Databases (http://www.gsea-msigdb.org/gsea/msigdb/index.jsp) and was used for the GO enrichment analysis (Powers et al., 2018). The FDR <0.1 was considered to be statistically significant. In addition, a single cell analysis was conducted based on the GSE146771 to seek more evidence on the potential function of *FSTL3* (Cao et al., 2021).

### 2.5 Immune Cell and Stromal Cell Analyses

The ssGSEA algorithm was initially performed to assess the correlation between the *FSTL3* expression levels and the overall immune as well as stromal infiltration levels in CRC (Liu et al., 2020). Furthermore, a high-performance computational method for quantifying cellular components from bulk tissue gene expression profiles, CIBERSORT was used to estimate immune infiltrations reliably (Chen et al., 2018). Spearman’s rank correlation coefficient was calculated for pairwise correlation comparisons and *p* < 0.05 was considered statistically significant. All the results from the above analyses methods and R package were implemented by the “ggplot2” and “pheatmap” packages.

### 2.6 Antibodies and Reagents

A complete list of reagents and antibodies is provided in Supplementary Tables. All the concentrations were chosen based on the previous studies or the manufacturer’s instructions. The experimental details are given in Supplementary material.

### 2.7 Cell Culture

The human CRC cell lines, SW620, SW480, RKO, HT-29, LoVo, Caco2, human monocytic cells THP-1 and normal human colonic epithelial cell line, NCM460 were purchased from the cell bank of the Chinese Academy of Sciences (Shanghai, China). The CRC and THP-1 cells were cultured in the RPMI-1640 medium with 10% fetal bovine serum (FBS). NCM460 was cultured in DMEM with 10% FBS. All the cells were incubated in 5% CO_2_ at 37°C.

### 2.8 Western Blot Assessment

The protocol for western blotting was based on the previous studies (Hnasko and Hnasko, 2015). The Target/β-actin bands were identified using a gel image processing system (ChemiDoc XRS+). Subsequently, the relative protein levels were calculated.

### 2.9 Ethics Statement and Specimen Collection

The study’s protocol was approved by the ethics committee of the Jiangsu Province Hospital of Chinese Medicine, and informed consent was obtained from clinicians and patients (2020NL-107-01). The CRC tissue and the adjacent healthy tissues (margin, 5 cm) were collected during surgery from 30 previously treatment-naïve patients with CRC at the Jiangsu Provincial Hospital of Traditional Chinese Medicine. The tumors were staged and graded using the 8th edition of the American Joint Committee on Cancer tumor-node-metastasis (TNM) staging system (Tong et al., 2018). After extraction, the tissue specimens were rinsed with cold phosphate-buffered saline and immediately placed in liquid nitrogen. The flash-frozen tissues were then transferred and stored at −80°C until further examination using immunohistochemistry (IHC) and western blot analysis. The preoperative serum samples were collected from all the patients, and serum was stored at −80°C for further studies.

### 2.10 Immunohistochemistry

The protocol used for IHC was based on earlier studies (Nizioł et al., 2021). The images were captured using a NIKON Eclipse Ni-E microscope (NIKON, Japan) (original magnification, ×400). The H-SCORE (range 0–300, higher scores indicating stronger positive staining) was calculated as described previously (Yang et al., 2019).

### 2.11 Lentiviral Vector Construction and Transfection

The lentiviral vectors were used for overexpressing and knocking down *FSTL3.* The viruses were designed, synthesized, and produced by the GeneChem Corporation. Transfection was performed according to the supplier’s protocol. The HT-29 and RKO cells were transduced with the recombinant lentivirus using 2 μg/ml polybrene for 24 h. Subsequently, the stably transfected GFP-expressing cells were identified using 1.5 μg/ml puromycin. The *FSTL3* overexpression and knockdown and transduction efficiency were assessed using western blots and GFP-expression.

### 2.12 CCK8 Assay

The CCK8 assay was performed using a CCK8 kit following the manufacturer’s protocol. Briefly, CRC cells were plated into 96-well plates (5 × 10^3^ cells per well) in 100 μl of culture medium or serum-free condition for 12, 24, 48 h at 37°C. CCK-8 solution (100 μl/well) was added for another 2 h and then incubated for 12, 24, and 48 h. Then, the optical density (OD) was measured at 450 nm with a microplate reader (BioTek Synergy HT).

### 2.13 Enzyme-Linked Immunosorbent Assay

The cell supernatants or patients’ serum was examined for FSTL3/FN1/α5β1 expression using the ELISA Kit based on the given instruction manual. A microplate reader (BioTek Synergy HT) was used to examine the optical density at 450 nm.

### 2.14 Colony Formation Assays

The clonogenic ability of the cells was assessed using a clone formation assay, performed as described previously (Grover et al., 2016). The number of colonies was counted using a compound light microscope (Olympus BX53, Japan).

### 2.15 Xenograft Tumor Model

All the animal experiments were approved by the ethics committee of the Jiangsu Province Hospital of Chinese Medicine (2021-5-062). Twenty-four 4-week-old male BALB/c nude mice were obtained from the Beijing Institute of Biomedicine (Beijing, China) (Certificate No. SYXK 2019-0010). The RKO cells transfected with sh-FSTL3, oe-FSTL3, and NC and control cells (4 × 10^6^ cell/mouse) were injected subcutaneously into the right armpit region (*n* = 6 per group). Seven days later, tumor formation was observed beneath the skin. The maximum 1) and minimum tumor diameter 2) were measured twice weekly. On day 28, the mice were euthanized and all the tumors were collected. The tumor volume was calculated (V = 1/2ab^2^), and the growth curves of the subcutaneous xenografts were drawn.

### 2.16 Wound Healing Assay

The protocol used for the wound healing assay was based on earlier studies (Han et al., 2020). Cell migration towards the scratch zone was photographed using an inverted fluorescence microscope (Olympus CKX-41, Japan) (×200 magnification).

Cells were dissociated to produce single-cell suspensions and were seeded in six-well ultralow-attachment plates at a density of 5 × 10 ^3^ cells/well. They were cultured in serum-free medium DMEM with FGF (20 ng/ml), EGF (20 ng/ml), and 2%B27. After culturing for 7 days, the size and number of tumor spheres were evaluated using light microscopy (Olympus BX53, Japan) (×40 magnification).

### 2.17 Transwell Assay

The cell migration and invasion were assessed using a transwell assay based on a previously published protocol (Wang et al., 2021). The membrane in the chamber was cut and imaged using light microscopy (Olympus BX53, Japan) (×200 magnification), and the cell counts were obtained using ImageJ software.

### 2.18 Immunofluorescence Staining

The protocol used for immunofluorescence staining was based on earlier studies (Donaldson, 2015). The immunofluorescence staining was observed using epifluorescence microscopy (Olympus, BX60-32FB2-A03) and different filters were used for capturing images using an Olympus, DP50 camera (×400 magnification).

### 2.19 Establishment of a Co-culture Unit

The THP-1 cells (1 × 10^5^ cells/ml) were treated with phorbol 12-myristate 13-acetate (PMA) (10 ng/ml) for 48 h to allow the induction of macrophage differentiation (Genin et al., 2015). The PMA-containing medium was replaced with the serum-free medium, and the cells were cultured for 24 h. Two days before the co-culture experiment, the cells (1 × 10^5^ cells/ml) from the control, knock-down (sh-*FSTL3*), overexpression (oe-FSTL3), and negative control (NC) groups were seeded onto the 0.4 μM transwell inserts. For co-culture, the culture medium in the inserts with the CRC cells was removed and transferred to the top of the pates with differentiated THP-1 cells. After 48 h of further co-culturing, the cells were obtained, and immunofluorescence staining was performed.

### 2.20 Statistical Analysis

Data were reported as mean ± standard deviation. The t-tests and one-way ANOVA were used to perform comparisons between the two groups and among the multiple groups, respectively. All data were analyzed using the SPSS 26.0 (SPSS Inc., USA) and illustrated using the GraphPad Prism 8.0 (GraphPad Software, Inc., USA). All the experiments were carried out at least thrice. **p < 0.01 and *p < 0.05 were defined to be statistically significant.

## 3 Results

### 3.1 Follistatin-Like 3 Expression in Colorectal Cancer

**FIGURE 1:**
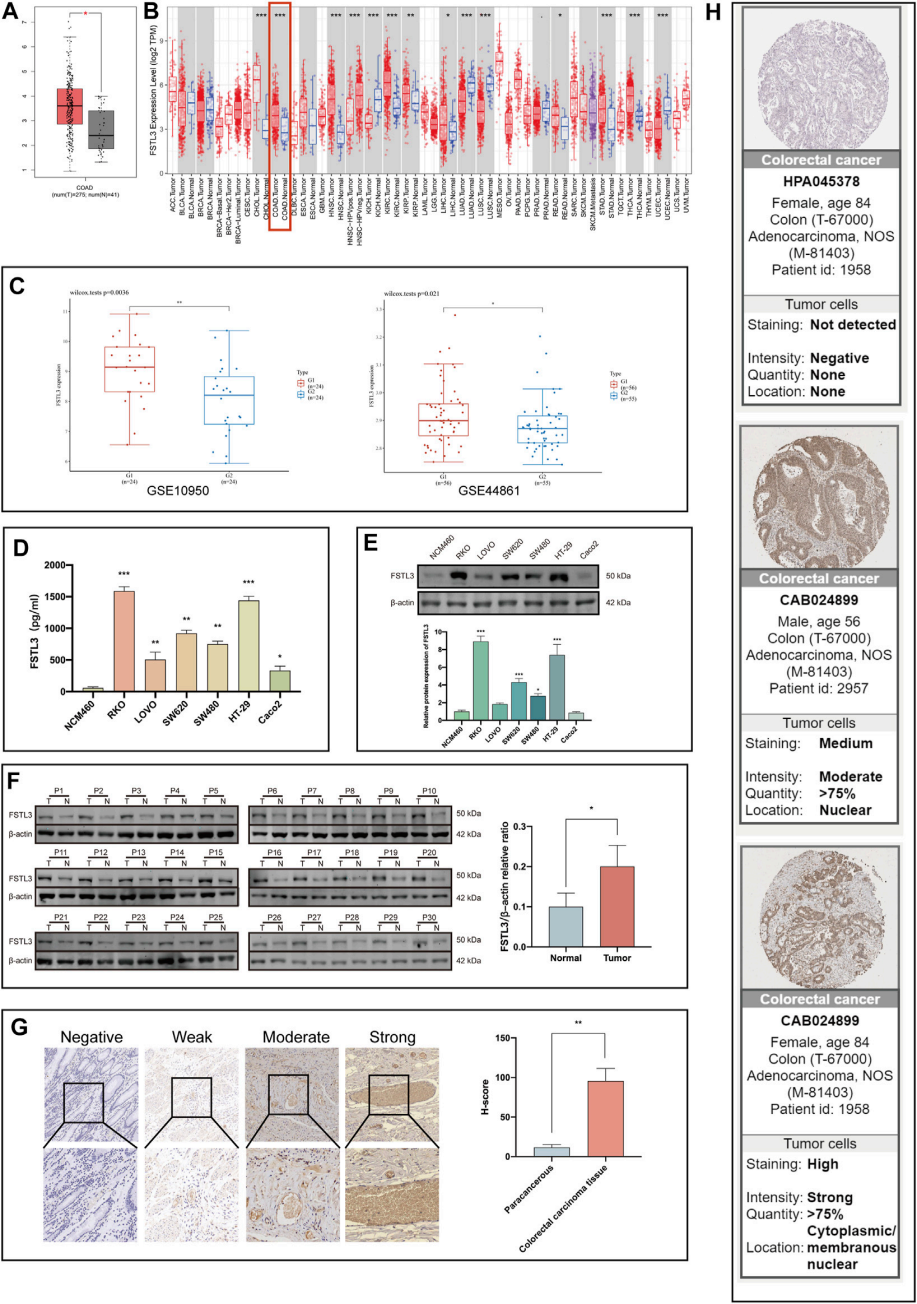
*FSTL3* levels in the colorectal cancer (CRC) tissues. **(A)** Expression levels of *FSTL3* in CRC based on the data of the Cancer Genome Atlas (TCGA)-COAD. **(B)** The *FSTL3* mRNA levels in the CRC and normal tissues based on the TIMER database. **(C)** Public datasets from the Gene Expression Omnibus (GSE10950, GSE44861) were used to verify the *FSTL3* mRNA levels in CRC. **(D,E)** The FSTL3 protein expression in the normal human colonic epithelial cells as well as the CRC cells. **(F)** FSTL3 expression in the CRC tissues (T) and paired non-tumorous tissue (N) evaluated using western blotting (*n* = 30). **(G)** The intensity of FSTL3 immunohistochemistry staining and FSTL3 expression levels in the paracancerous and CRC tissues (*n* = 30). **(H)** The FSTL3 immunohistochemistry in the CRC tissues is based on data from the Human Protein Atlas (THPA). * *p* < 0.05, ** *p* < 0.01, *** *p* < 0.001.

The TCGA-COAD data showed that the *FSTL3* expression is higher in the CRC tissues than in the normal tissues (Figure 1A; *p* < 0.05). Moreover, the TIMER and TCGA data analyses also showed higher *FSTL3* expression in the CRC than in the normal tissues (Figure 1B).

The expression of *FSTL3* was further explored in the CRC tissues using data from the Gene Expression Omnibus (GEO) database. Data from the GSE10950 and GSE44861 datasets indicated a significant difference in the FSTL3 expression between the CRC tissues and adjacent tissues (Figure 1C). Western blot, ELISA, and IHC staining revealed that FSTL3 was overexpressed in the CRC cells and tissues. The mean H-SCOREs for FSTL3 expression in the CRC and paracancerous tissues were 95.53 ± 15.96 and 11.70 ± 3.55, respectively (Figures 1D–G) (*p* < 0.01, ANOVA). The FSTL3 protein expression in CRC was further verified using the IHC data from The Human Protein Atlas, which revealed that FSTL3 to be primarily expressed in the cell membrane and cytoplasm (Figure 1H).

### 3.2 Prognostic Value of the Follistatin-Like 3 Expression in Colorectal Cancer

**FIGURE 2:**
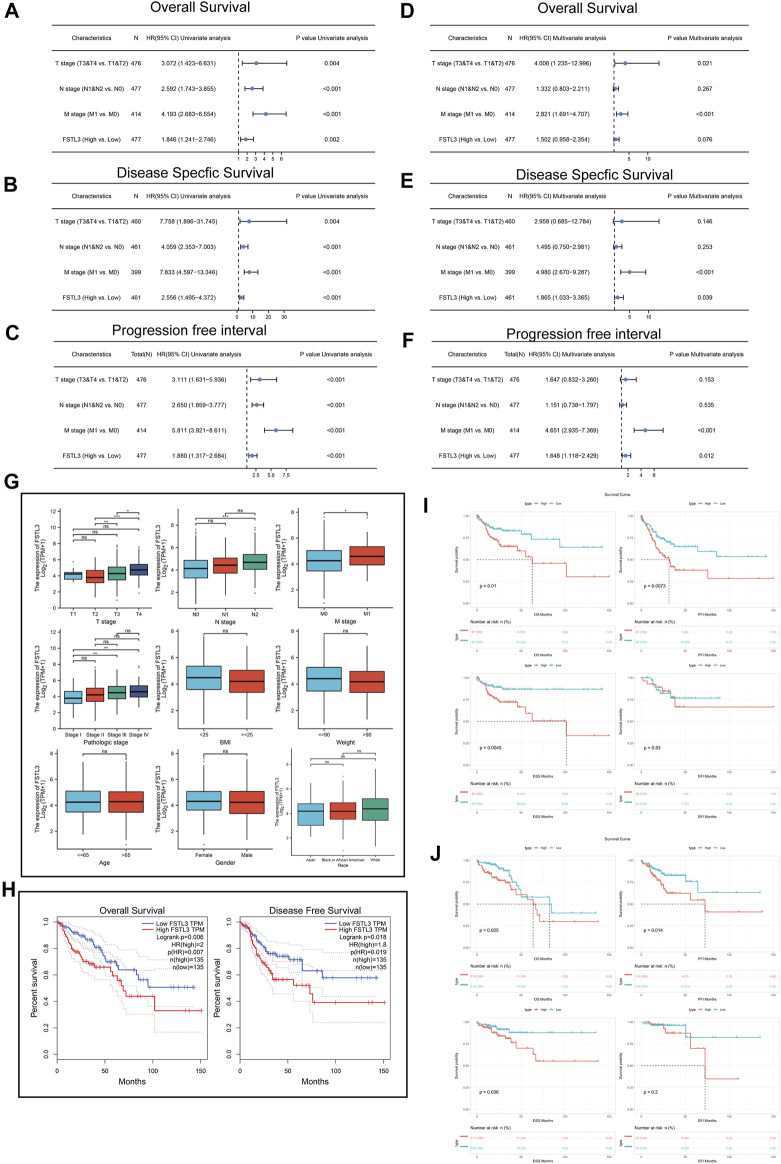
Univariate and multivariate Cox regression analyses of *FSTL3* expression and clinicopathological factors related to the prognosis of colorectal cancer (CRC). **(A–C)** Univariate Forest plot illustrating *FSTL3* expression and the clinicopathological factors related to the **(A)** Overall Survival (OS), **(B)** Disease-Specific Survival (DSS), and **(C)** Progression Free Interval (PFI) in CRC. **(D–F)** Multivariate Forest plot showing *FSTL3* expression and the clinicopathological factors related to **(D)** OS, **(E)** DSS, and **(F)** PFI in CRC. **(G)** Association of the *FSTL3* mRNA expression with the T/N/M stage, pathological stage, BMI, weight, age, sex, and race. **(H)** Overall survival (OS) and Disease-free survival (DFS) from the GEPIA database. **(I,J)** The OS, disease-free interval (DFI), DSS, and PFI from The Cancer Genome Atlas-COAD database based on the *FSTL3* expression levels. **(I)** KRAS mutated; **(J)** KRAS wild-type. Hazard ratios and *p*-values are shown. NS: not significant, * *p* < 0.05, ** *p* < 0.01, *** *p* < 0.001.

The multivariate hazard ratios for the different variables were then calculated using a Cox regression model based on the TCGA-COAD. Univariate analysis results (OS) demonstrated that the *FSTL3* overexpression (*p* = 0.002), T classification (*p* = 0.004), N classification (*p* < 0.001), and M classification (*p* < 0.001) were all closely correlated with a poor prognosis (Figure 2A). The multivariate analyses revealed that the T classification (*p* = 0.021) and M classification (*p* < 0.001) were all independent predictors of an unfavorable prognosis (Figure 2D).

Furthermore, the results of the univariate analysis (DSS) demonstrated that *FSTL3* overexpression (*p* < 0.001), T classification (*p* = 0.004), N classification (*p* < 0.001), and M classification (*p* = 0.004) were in close correlation with a poor prognosis (Figure 2B). The multivariate analyses revealed soverexpression (*p* = 0.039), and M classification (*p* < 0.01) to independently predict unfavorable prognosis (Figure 2E).

Finally, the results of the univariate analysis (PFI) demonstrated *FSTL3* overexpression (*p* < 0.001), T classification (*p* < 0.001), N classification (*p* < 0.001), and M classification (*p* < 0.001) to be closely correlated with a poor prognosis (Figure 2C). The multivariate analyses revealed *FSTL3* overexpression (*p* = 0.012), and M classification (*p* < 0.001) to independently predict the unfavorable prognosis (Figure 2F).

The *FSTL3* levels are related to the clinicopathological characteristics of the CRC patients, including the T/N/M stage, pathological stage, BMI, weight, age, sex, and race, as illustrated in Figure 2G.

To evaluate the association of *FSTL3* expression levels with the survival in CRC patients, the GEPIA database was used. Interestingly, the expression of FSTL3 showed a significant negative correlation with the patient survival (GEPIA: Overall survival (OS), *p* = 0.006; Disease-Free Survival (DFS), *p* = 0.018) (Figure 2H).

The results based on the TCGA-COAD revealed the increasing FSTL3 expression levels to be associated with a worse prognosis (OS, *p* = 0.01; PFI, *p* = 0.0073; DSS, *p* = 0.0045) in the KRAS wild type (Figure 2I) and mutant type (Figure 2J).

### 3.3 Functional Enrichment Analysis of Follistatin-Like 3

**FIGURE 3:**
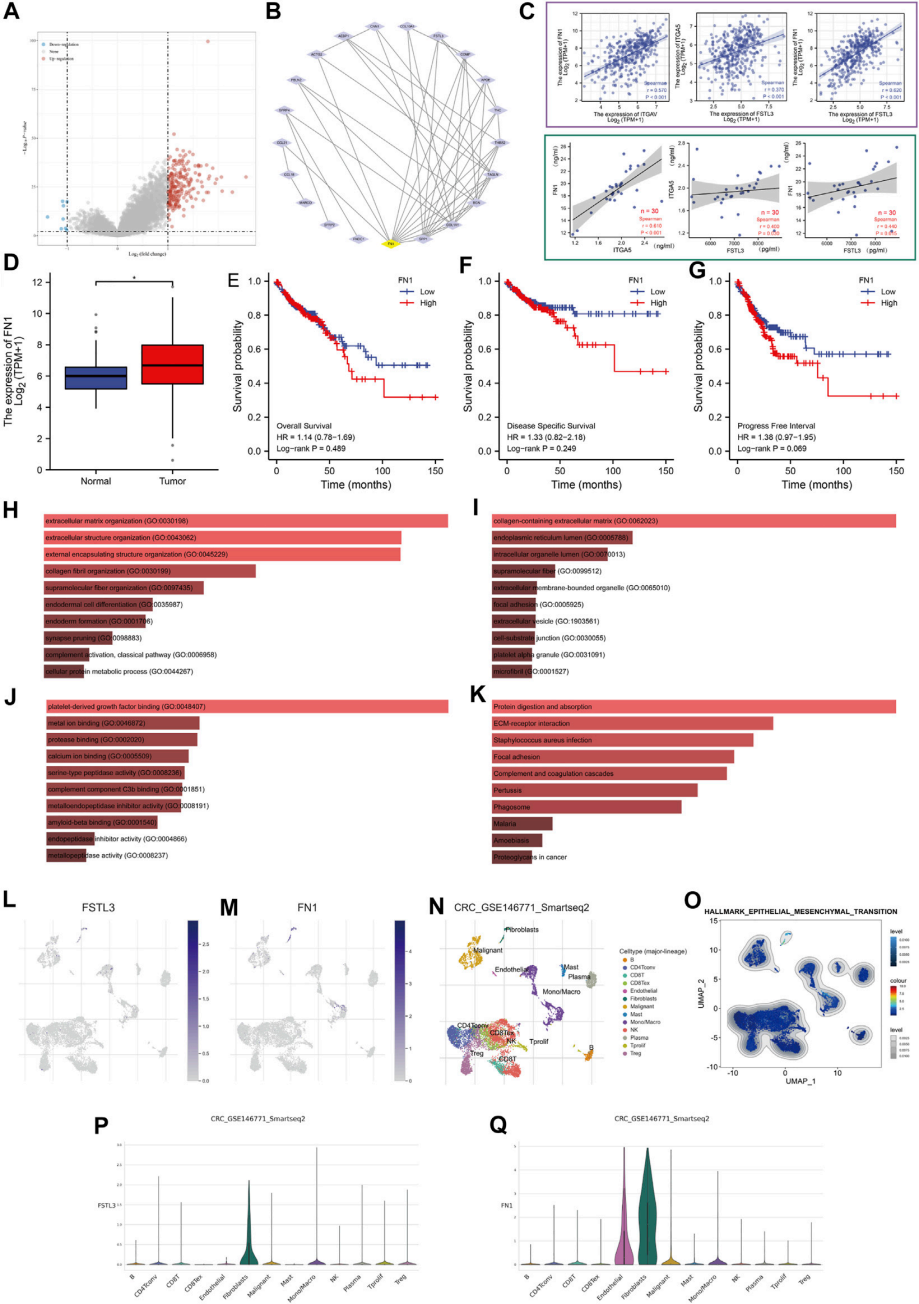
Protein-protein interaction (PPI) network and enrichment analysis. **(A)** Volcano map of the genes showing differential expression after a change in the *FSTL3* levels. Red dots, upregulated genes; blue dots, downregulated genes; abscissa, differences in the gene expression (log2 fold change); and ordinate, the significance of these differences (−log10 padj). **(B)** The protein-protein interaction (PPI) network of *FSTL3* and its related genes. **(C)** TCGA-based correlation analysis between *FN1, ITGA5,* and *FSTL3* (area within the green box). Analysis of the correlation between the serum FN1, ITGA5, and serum FSTL3 in the patients based on the comparison between the two groups (area within the purple box). **(D)** Expression of *FN1* in the normal and tumor tissues based on TCGA-COAD data. **(E–G)** The OS, DSS, and PFI from The Cancer Genome Atlas-COAD database are based on the FN1 expression levels. **(H–K)** Functional enrichment analysis of the *FSTL3*-related genes. **(H)** Biological Processes (BP), (I) Cellular Components (CC), **(J)** Molecular Functions (MF), and **(K)** Kyoto Encyclopedia of Genes and Genomes (KEGG).**(LM)** Uniform manifold approximation and projection (UMAP) plots illustrating the expression of (L) *FSTL3* and (M) *FN1* clusters. **(N)** UMAP plots illustrating the CRC cell landscape. We found 13 cell types across all cells after quality control, dimensionality reduction, and clustering. **(O)** Enrichment score for the genes from the hallmark hypoxia gene set in each cell was obtained using the gene set variation analysis. **(P,Q)** Violin plots for CRC cell cluster marker genes and **(P)** *FSTL3*, **(Q)** *FN1* in different cell types. Expression was measured as log 2 (TP10K + 1). NS: not significant, * *p* < 0.05, ** *p* < 0.01, *** *p* < 0.001.

The genes positively or negatively correlated with *FSTL3* in TCGA-COAD were obtained based on the “DESeq” R package (Figure 3A). A total of 21 differentially expressed genes (DEGs) were imported into the DEGs PPI, and then the Cytotype cytoHubba was applied for further analysis demonstratingFN1 to be the highest-scoring hub gene (Figure 3B). The correlation analyses between the serum FN1, ITGA5 (gene symbol for α5β1), and serum FSTL3 in the patients by comparing between the two groups (area within the purple box) and TCGA-COAD-based correlation analyses between *FN1, ITGA5,* and *FSTL3* (green box). The results showed a highly significant positive correlation between the expression of FSTL3 and FN1 and ITGA5 (Figure 3C). FN1 was overexpressed in COAD (Figure 3D) but was not correlated with the prognosis of COAD patients (Figures 3E–G). The analysis of the functional enrichment showed *FSTL3* to be involved in “extracellular matrix organization,” “extracellular structure organization,” “collagen-containing extracellular matrix,” “platelet-derived growth factor binding,” “Protein digestion and absorption,” ECM-receptor interaction,” and “Focal adhesion.” Most results have been correlated to the EMT or extracellular matrix (ECM) leading to the invading phenotype of the carcinoma cells. (Figures 3H–K).

In the single-cell level study, the *FSTL3* and *FN1* were more inclined to express on the fibroblasts (Figures 3L–Q), which are important players in the EMT.

### 3.4 Relationship Between Follistatin-Like 3 and Epithelial-Mesenchymal Transition and Its Underlying Mechanism

**FIGURE 4:**
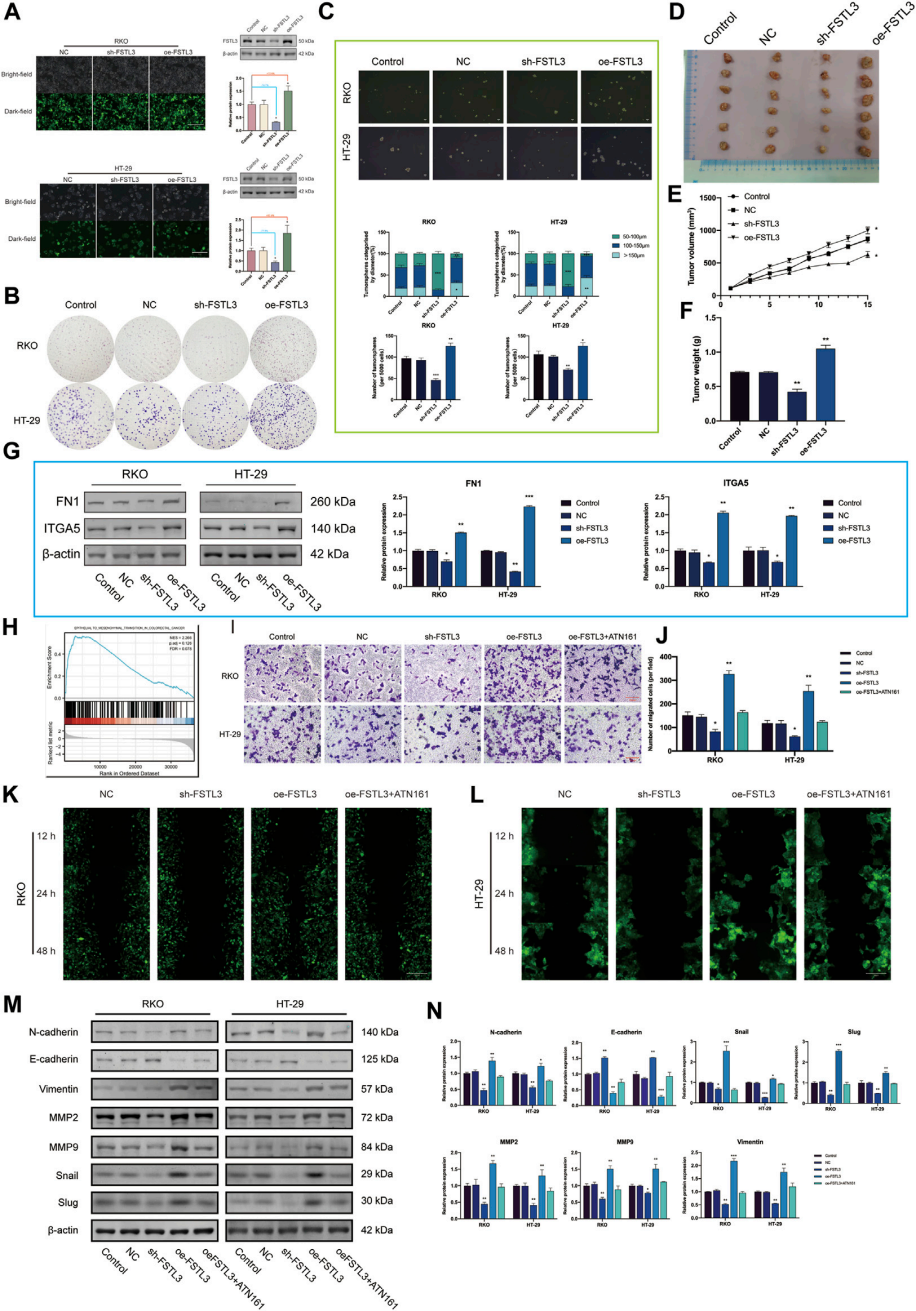
*FSTL3* overexpression promotes a malignant phenotype in colorectal cancer (CRC). **(A)** The transfection efficiency was verified by the GFP expression as well as the western blot. Transfection efficiency is represented as percentages. **(B)** Clone formation capacity of the CRC cells transfected with the NC, sh- *FSTL3*, and oe-*FSTL3* constructs assessed using the clone formation assay. **(C)** Representative images from the sphere-forming assay. The number of tumorspheres was counted and plotted, and percentage of tumorspheres with diameters of 50–100 μm, 100–150 μm, or >150 μm was calculated and plotted. The scale bar represents 100 μm. **(D)** The xenograft tumors from the nude mice. **(D–F) (E)** Tumor volume and **(F)** weight of xenografts from the nude mice. (G) FN1, ITGA5 levels in CRC cells transfected with NC, sh-*FSTL3*, and oe-*FSTL3* were examined using western blot. **(H)** Gene Set Enrichment Analysis (GSEA) of *FSTL3.* **(I,J)** The invasion ability of CRC cells after transfection; the relative invasive cell number is shown towards the right. **(K,L)** The migratory ability of the different groups of CRC cells [**(K)**: RKO, **(L)**: HT-29] was examined using the wound-healing assays. **(M,N)** Expression of the EMT-related proteins was examined using western blots after the transfection of the CRC cells with NC, sh- FSTL3, and oe-FSTL3 constructs and treatment with 10 μM ATN-161, an antagonist of integrin α5β1. The statistical analysis of the western blot result is shown towards the right. * *p* < 0.05, ** *p* < 0.01, *** *p* < 0.001.

The expression level of FSTL3 was the highest in the RKO and HT-29 cell lines, therefore these were selected for further experiments. The transfection efficiency was verified by GFP expression and western blot ((Figure 4A) (*p* < 0.01). *FSTL3* (either overexpression or knockdown) does not have an obvious effect on cell proliferation as well as cell viability under either with serum or without serum condition for 48 h (Supplementary Figure S3). *FSTL3* silencing decreased the capacity of forming the tumor cell clone as well as the sphere-forming abilities (Figures 4B,C). Moreover, the stable *FSTL3* overexpression in the RKO cells promoted the formation of the subcutaneous xenograft tumors *in vivo* (Figures 4D–F) (*p* < 0.01). Based on the TIMER, the data, and patient’s serum (*n* = 30) in Figure 3 C was the FSTL3 expression was positively correlated with that of *FN1,* and *ITGA5.* Subsequent the *in vitro* experiments using the western blot revealed a reduction in the levels of FN1, ITGA5 after *FSTL3* silencing (*p* < 0.05) (Figure 4G), and the opposite trend was observed when *FSTL3* was overexpressed. The GSEA for *FSTL3* revealed the potential role of *FSTL3* in “epithelial-mesenchymal transition in CRC” (Figure 4H). Based on this result, the EMT markers and EMT-related phenotypes were studied and the *FSTL3* overexpression was found to increase the EMT-related phenotypes. However, this effect was attenuated after treatment with the integrin ITGA5 -specific inhibitor, ATN-161 (Figures 4I–N). The cell viability assays showed the cell proliferation to be unaffected by the selected concentration of ATN-161 (Supplementary material).

### 3.5 Relationship of the Follistatin-Like 3 Expression With the Cytoskeletal Remodeling in the Colorectal Cancer Cells

**FIGURE 5:**
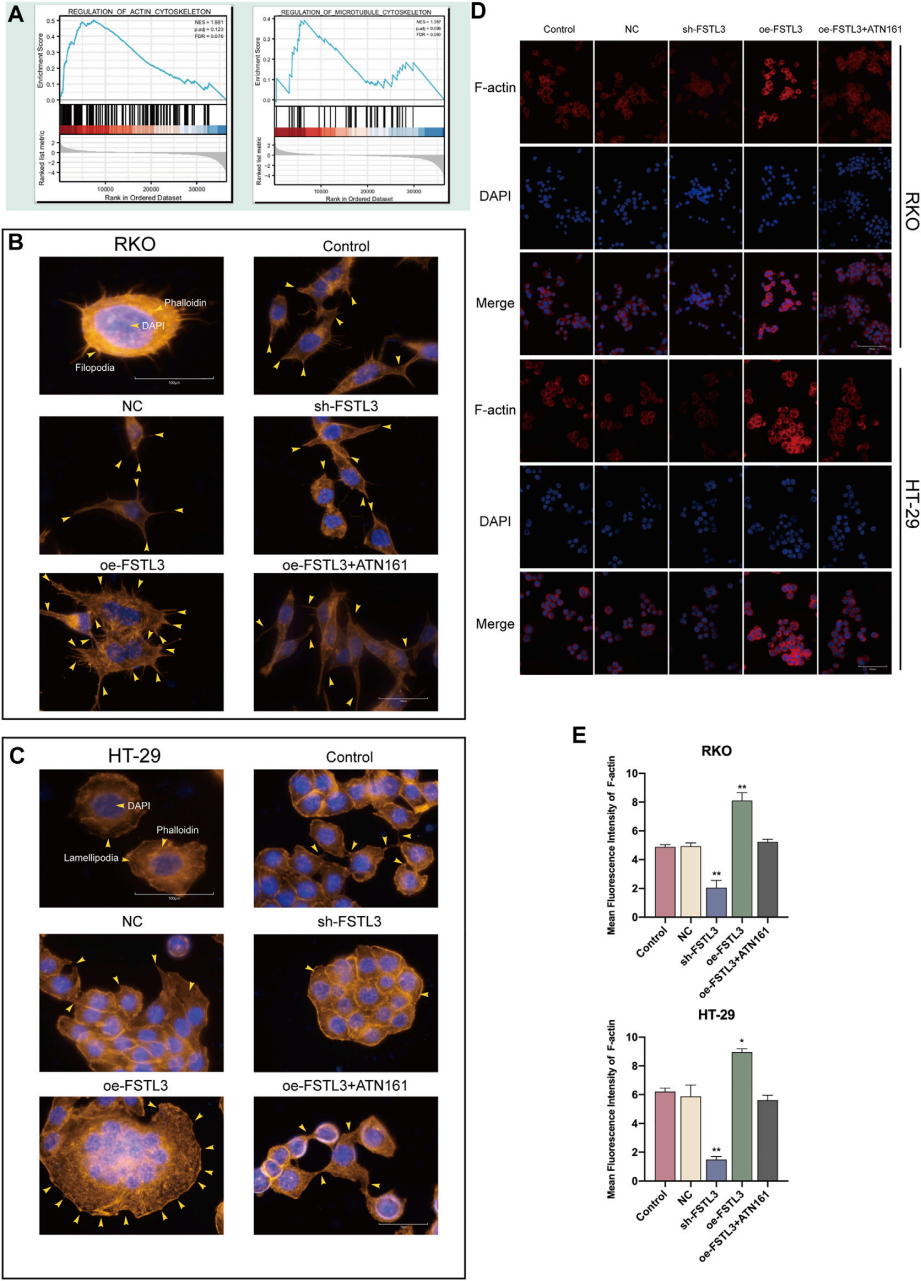
Relationship between the *FSTL3* and the F-actin cytoskeleton. **(A)** Gene Set Enrichment Analysis (GSEA) of *FSTL3*. **(B–D)** The cytoskeleton of colorectal cancer (CRC) cells (control cells and CRC cells transfected with the NC, sh-*FSTL3*, and oe-*FSTL3* constructs) treated with 5 μM ATN-161 detected using phalloidin staining and immunofluorescence staining. [**(B)**: RKO, **(C)**: HT-29] Yellow arrowheads point to filopodia/larger lamellipodia/pseudopodia-like protrusions. **(E)** Immunofluorescence intensities expressed as mean intensity ±SD. * *p* < 0.05, ** *p* < 0.01, *** *p* < 0.001. Arrowheads indicate pseudopodia.

GSEA also revealed the functional enrichment for *FSTL3* under the “regulation of actin cytoskeleton” and “regulation of microtubule cytoskeleton” domains (Figure 5A). Subsequent *in vitro* experiments revealed that *FSTL3* overexpression makes the cell pseudopodia longer and more obvious (Figures 5B,C) and the upregulation of F-actin (Figures 5D,E). Consistent with the prior experiments, this effect was attenuated after treatment with ATN-161 (*p* < 0.01).

### 3.6 Relationship of Follistatin-Like 3 Expression With the M2 Macrophage Infiltration

**FIGURE 6:**
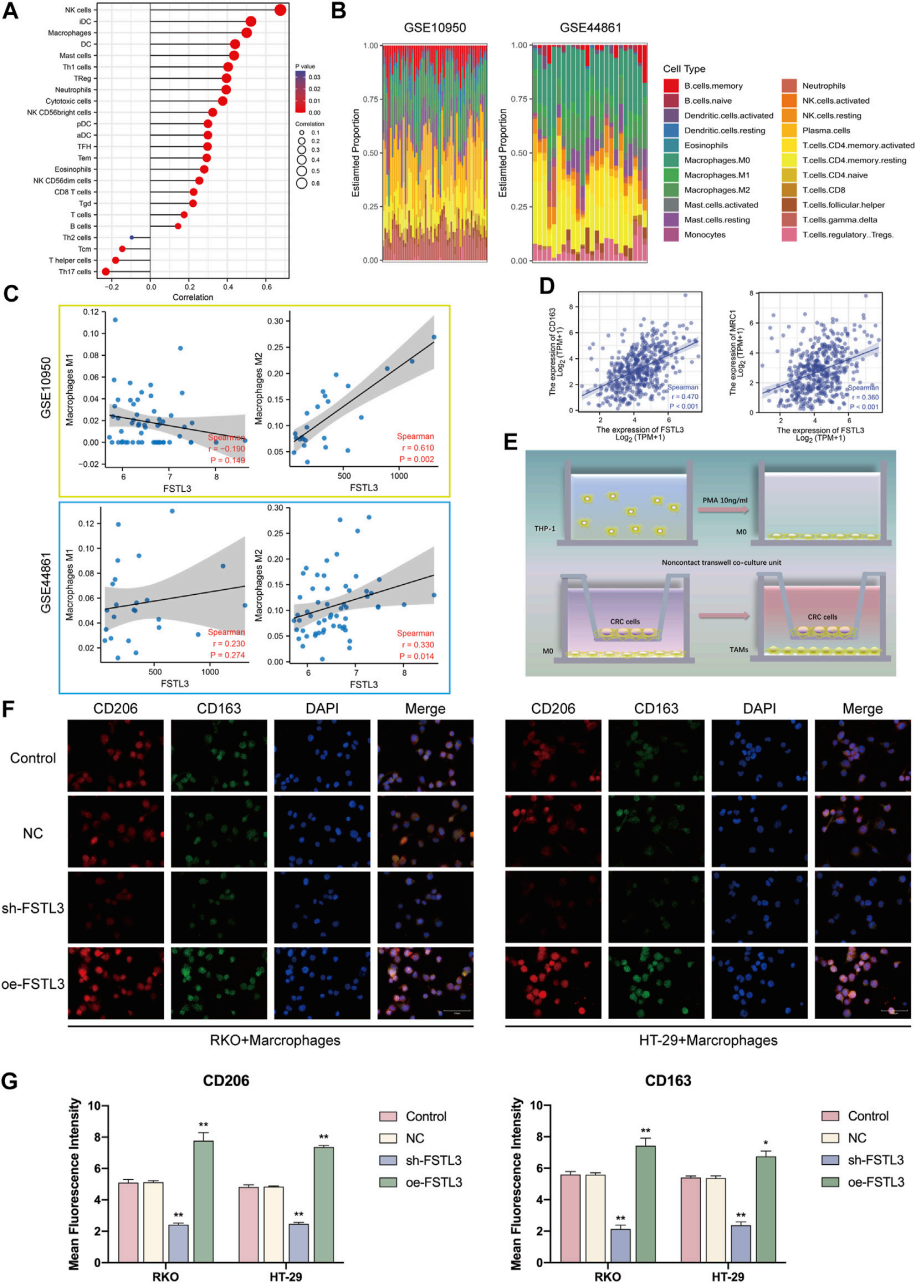
Immune landscape of the colorectal cancer (CRC) patients with the different expression levels of *FSTL3* and the relationship between the *FSTL3* expression and the abundance of M2 tumor-associated macrophage infiltration. **(A)** Spearman correlation between *FSTL3* and 24 types of immune cells; positive correlation, red lollipop, and negative correlation, blue lollipop. **(B)** Proportion of 22 types of Tumor-initiating cells (TICs) in the CRC tumor samples based on GSE10950 and GSE44861. **(C)** Correlation of the *FSTL3* expression levels with macrophage abundance based on GSE10950 and GSE44861. **(D)** Correlation of the *FSTL3* expression levels with M2 macrophage markers. **(E)** Schematic diagram for the tumor–macrophage cell co-culture. **(F)** Immunofluorescence staining for CD206 (red) and CD163 (green). **(G)** Immunofluorescence intensities are expressed as mean intensity ±SD. * *p* < 0.05, ** *p* < 0.01, *** *p* < 0.001.

The ssGSEA algorithm was used to calculate the correlation between the *FSTL3* expression and infiltration degree of several immune cells (Figure 6A). Considering *FSTL3* to be associated with the abundance of macrophages, the relationship between the *FSTL3* and macrophage abundance was further assessed using the GSE10950 and GSE44861 datasets. After excluding the normal samples from these two datasets, 22 immune cell profiles were obtained for the CRC samples (Figure 6B). Subsequently, the GSE10950 and GSE44861 datasets were used to assess the relationship between the *FSTL3* expression and the macrophage infiltration The *FSTL3* expression levels were positively correlated with the M2 macrophage abundance (Figure 6C). Therefore, the correlation between the *FSTL3* and the M2 surface markers were calculated using the TIMER database and a positive correlation was observed between the *FSTL3* expression and *MRC1* (CD206) (R = 0.36, *p* < 0.001) and *CD163* (R = 0.47, *p* < 0.001) expression (Figure 6D). This series of results suggested a positive association between the *FSTL3* expression and M2 macrophage infiltration. To further investigate the influence of *FSTL3* overexpression on the M2 macrophage abundance in CRC, a tumor–macrophage cell co-culture model was established using a transwell non-contact co-culture unit (Figure 6E). The *FSTL3* overexpression was found to significantly upregulate the surface markers of the M2 tumor-associated macrophages (TAMs) (CD206 and CD163) (Figures 6F,G).

Hence, the findings of this study confirmed the *FSTL3* levels to be positively correlated with the abundance of M2 macrophage infiltration.

## 4 Discussion

The CRC patients demonstrate high incidence and poor prognosis due to postoperative metastasis and local recurrence (Dekker et al., 2019). Nearly half of the CRC patients die within 5 years from diagnoses (Kumar et al., 2021). CRC is a highly complex and heterogeneous disease evolving not from the dysfunction of a single gene but the synergistic behavior of numerous genes in a complex network (Denlinger and Barsevick, 2009). This necessitates the identification of the key genes involved in this network. It can, therefore, provide new ideas for the targeted therapy and assessment of prognosis. The previous studies of our group have demonstrated a coactivating relationship between *FSTL3* and the BMP/SMAD signaling, showing that it can regulate the SMAD phosphorylation and promote EMT in gastric cancer cells (Liu et al., 2021). Since *FSTL3* is a potentially useful target gene for tumor therapy, it not only directly affects the biological characteristics of the tumor cells but also remodels the tumor microenvironment (TME) and influences the prognosis of the patient.

The extracellular matrix (ECM) is a protein scaffold defining a part of the extracellular microenvironment and forming the non-cellular component of the cancer tissues (Pickup et al., 2014). ECM is associated with a variety of functions, including mechanical support and biochemical signaling (Theocharis et al., 2016). ECM degradation is considered the first step in cancer invasion and metastasis (Bonnans et al., 2014). Therefore, the tumor therapeutic strategies targeting the ECM are being increasingly developed and utilized at present (Abyaneh et al., 2020). The results of the enrichment analysis suggested a possibly close link between *FSTL3* and ECM stating that *FN1* is a core factor in the FSTL3-related network. Fibronectin-1 (FN1) is a large ECM protein with an important role in cell adhesion, cell migration, invasion, EMT as well as TME, mediated through integrin signaling (Efthymiou et al., 2020). Integrins are the heterodimeric cell surface glycoprotein receptors with 2 non-covalently -associated subunits linking the ECM to the intracellular cytoskeleton (Barczyk et al., 2010). They mediate the cell-cell and cell-matrix adhesion (Huttenlocher and Horwitz, 2011). The binding of integrins to FN1, transduce signals to the intracellular interior, with simultaneous reception of intracellular signals that regulate their ligand-binding affinity (Giancotti and Ruoslahti, 1999). Integrin α5β1 is a well-known major receptor for FN1(Miroshnikova et al., 2017) and was demonstrated to bind to its receptor integrin α5β1 activating the PI3K/Akt signaling pathway, thus, promoting the progression of breast cancers (Veevers-Lowe et al., 2011). Based on the results of the enrichment analysis, a single-cell level analysis was performed suggesting that *FSTL3* and *FN1* are predominantly expressed on the fibroblasts, inducing the production of the ECM components. Integrins are the key mediators of the cell–ECM interaction, linking the ECM to the actin cytoskeleton (Kechagia et al., 2019). FSTL3 overexpression has been subsequently demonstrated experimentally to significantly upregulated the FN1 and α5β1 expression. Then, the rhodamine staining was used to visualize the F-actin showing that the FSTL3-overexpressed cells tended to have more pseudopods. The results also showed that *FSTL3* overexpression upregulates the F-actin participating in the cytoskeletal remodeling in the CRC cells.

Cytoskeletal remodeling is involved in the EMT process, constituting the reorganization and reconstruction of the actin cortical cytoskeleton, which is an important hallmark of EMT (Yilmaz and Christofori, 2009). The cross-talk between FN1 and α5β1 triggers a cellular architectural change, with protrusion of different structures such as the pseudopodia, filopodia, or lamellipodia (Revach et al., 2020). We observed that FSTL3 upregulation significantly promoted the EMT phenotype, yielding cells with greater invasive capacity. As a proof-of-concept experiment, the integrin α5β1 specific inhibitor was used, which rescued the EMT phenotype and cytoskeleton remodeling caused by *FSTL3* overexpression.

The advent of immunotherapy using immune checkpoint (ICP) inhibitors in recent years has indicated the evaluation of the tumor immune microenvironment (TIME) landscape heterogeneity and reshaping TIME as promising approaches for prospective CRC treatment (Li et al., 2019). The tumor-associated macrophages (TAMs) are important components of the TIME (Pathria et al., 2019), mainly derived from circulating monocyte populations, exhibiting characteristics similar to that of the M2 macrophages (Yamaguchi et al., 2016). The M2 macrophages are known to be involved in ECM remodeling, angiogenesis, and immunosuppression (Najafi et al., 2019). Nearly 70% of the patients receiving ICP inhibitors therapy are non-responders or quickly attain drug resistance, primarily due to the M2 macrophage infiltration. Recent studies have found the M2 macrophages to express high levels of TGF-β1, promoting the ECM deposition and EMT (Liu et al., 2018; Zeng et al., 2019). However, the cancer cells undergoing EMT also promote the M2 macrophage infiltration by secreting the tumor metabolites (Dongre et al., 2017), forming a vicious circle. Analysis of the TCGA-COAD data by the ssGSEA algorithm showed a significant positive correlation between the expression level of *FSTL3* and the level of macrophage infiltration. Further calculations indicated an excellent correlation between *FSTL3* and the M2 macrophages. Furthermore, the macrophages with the CRC cells were co-cultured and immunofluorescence staining indicated the CRC cells to overexpress *FSTL3* with a greater ability to induce the M2 macrophage proliferation. These results revealed an important role for FSTL3 in remodeling the CRC TIME.

To sum up, the results of the study indicated that *FSTL3* is increased in CRC cells and tissues and that a high *FSTL3* level is related to the clinicopathological features and poor prognosis of colorectal cancer. These results suggest *FSTL3* be an important player in the complex gene regulatory mechanisms triggering CRC through the processes such as promoting the binding of FN1 to α5β1, activating the EMT phenotype, and M2 macrophage infiltration, ultimately promoting tumor progression. We initiated animal experiments after *in vitro* validation of the cell phenotype. The subcutaneous graft tumour model was chosen to allow more visualisation of the changes in tumour size, a higher rate of tumour formation and easy measurement of tumour changes. In fact, during the final animal autopsy, we found that some mice in the NC group, control group and the oe-*FSTL3* groups had liver metastases (scattered and different in size), which were not quantified for statistical comparison because the sample size was too small (Supplementary Figure S4). Therefore, based on the results of this animal study in which some mice developed liver metastases, we will use the venous metastatic tumour model, a model of tumour formation in one or more organs of the animal after injection of tumour cells into the tail vein, in our subsequent study of *FSTL3* on the tumour stromal microenvironment. This is more appropriate for studying metastasis *in vivo*. The use of Luciferase labelling of tumour cells in combination with *in vivo* imaging allows a clearer study of the effect of *FSTL3* on metastasis *in vivo*. In addition, there are some limitations to this study. The *in vivo* studies are still in infancy and most of the data analyzed in this study are derived from online databases and that the clinical sample size for this study was small, hence, further larger sample studies are necessary to substantiate the findings. In conclusion, the findings of this study provided exciting novel clues which require further focused studies for the elucidation of the mole.

## Data Availability

The datasets presented in this study can be found in online repositories. The names of the repository/repositories and accession number(s) can be found in the article/Supplementary Material.
