# Supplementary material for: Bioinformatic Analyses and Experimental Verification Reveal that High FSTL3 Expression Promotes EMT via Fibronectin-1/α5β1 Interaction in Colorectal Cancer
Source: Front Mol Biosci. 2021 Nov 24;8:762924. doi: 10.3389/fmolb.2021.762924 (PMC8652210; doi:10.3389/fmolb.2021.762924)
Supplement: Supplementary file 1 [file DataSheet1.PDF]

Supplementary Material

1 Supplementary figures

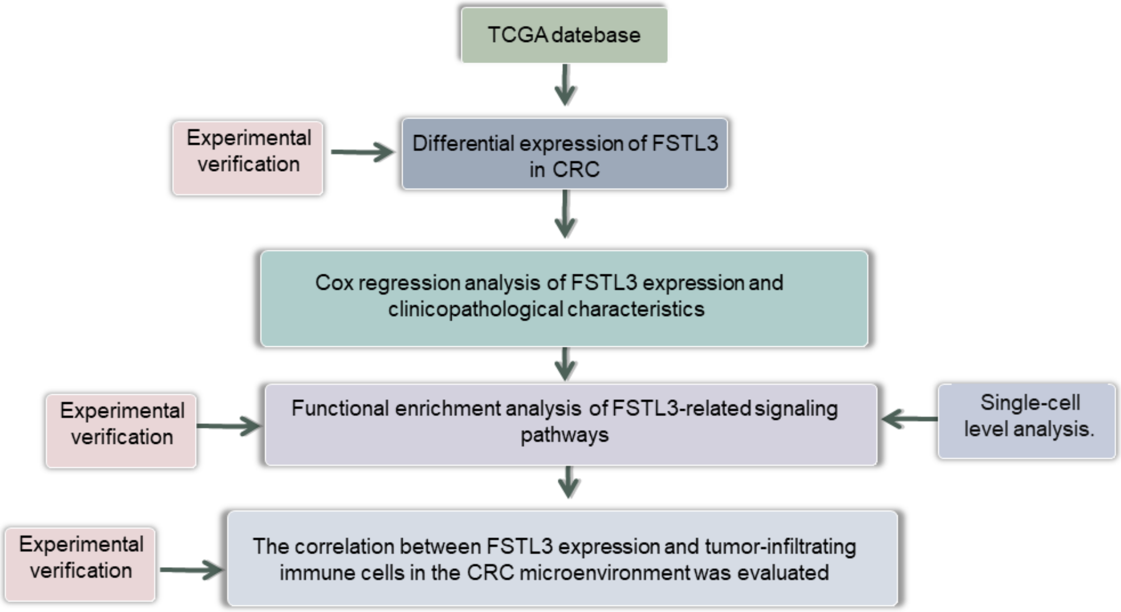

Supplementary Figure 1. Process flow of the study.

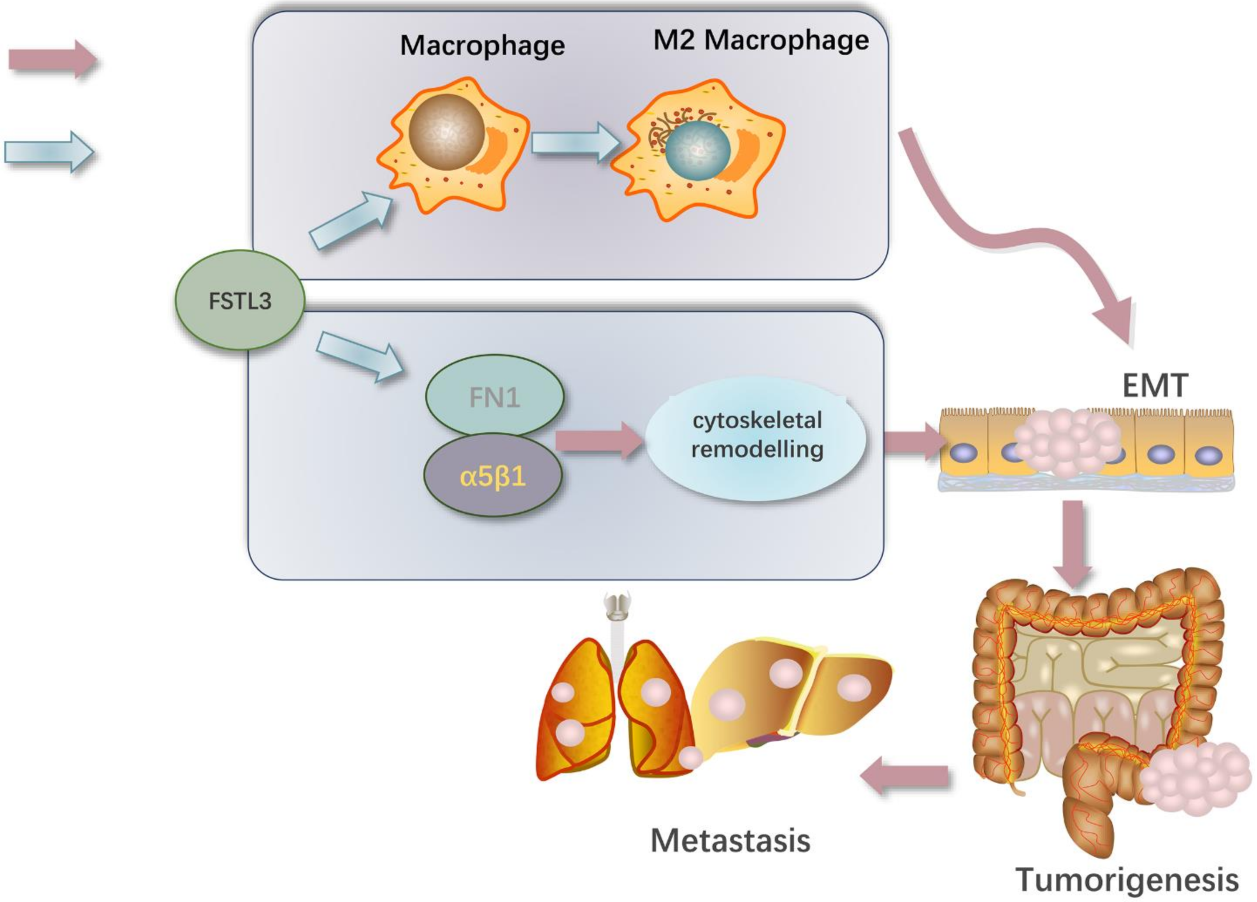

Supplementary Figure 2. Diagram depicting the mechanism of FSTL3 regulation in the tumorigenesis of colorectal cancer (CRC).

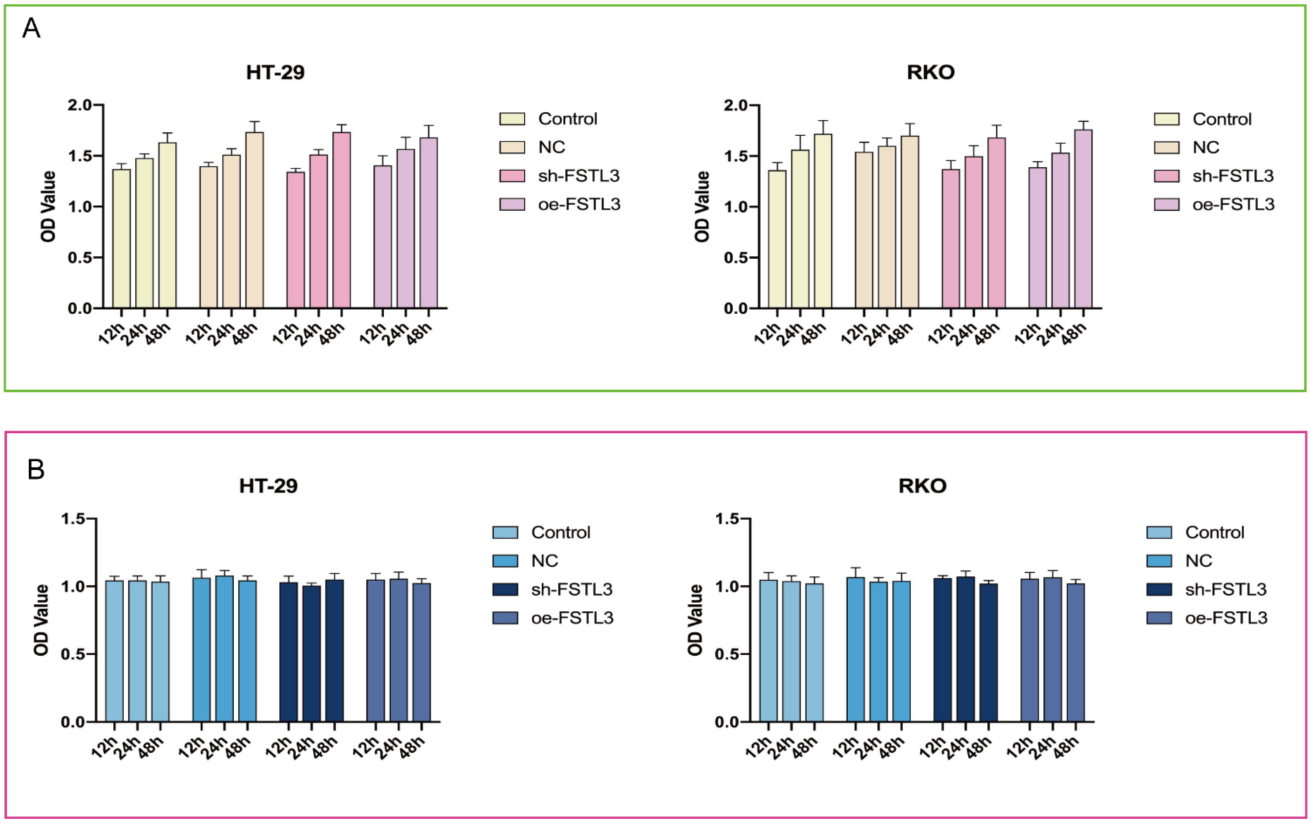

Supplementary Figure 3. CCK8 assay showed the optical density (OD) value of HT-29/RKO cells under 10 % serum (A) or serum-free (B) conditions.

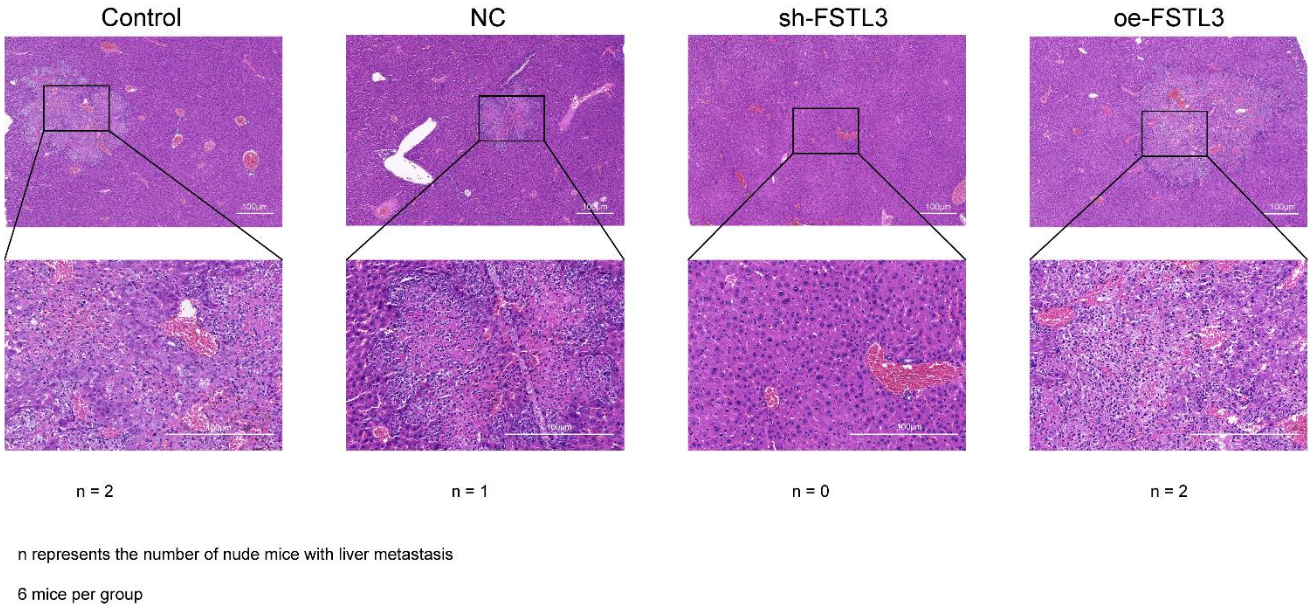

Supplementary Figure 4. Hematoxylin and eosin (HE) staining of liver sections

2 Supplementary Table

Antibodies and Reagents	Manufacturer, Country, Cat number	Concentration
Roswell Park Memorial Institute 1640 (RPMI-1640)	Gibco, USA, Lot: 8121248	-
Dulbecco's modified Eagle's medium (DMEM)	Gibco, USA, Lot: 8121032	
Fetal bovine serum (FBS)	Gibco, USA, Lot: 42F1376K	-
Lentiviral vectors of FSTL3	GeneChem, China, Lot: 136427D	-
Recombinant Human EGF Animal-Free manufactured	Peprotech, USA. Lot: 111908	20ng/ml
Recombinant Human FGF-basic (154 a.a.)	Peprotech, USA. Lot: 0820AFC05	20ng/ml
B27 supplement	Gibco, USA, Lot:2226816	2%
Polybrene	GeneChem, China. Lot: 139146B	2 μg/mL
Puromycin	Beyotime Biotechnology, China, Lot: 041321210517	1.5 μg/mL
Anti-FSTL3 Antibody	Invitrogen, USA. Lot: SI2436032	WB: 1:1000<br>IHC: 1:200
Human Fibronectin 1 (FN-1) ELISA Kit.	Aifang Biotechnology, China, Lot: J11064	-
Human ITG α5β1 (ITG α5β1) ELISA Kit	Aifang Biotechnology, China, Lot: J3051	-
Human Follistatin Like Protein 3 (FSTL3) ELISA Kit	Aifang Biotechnology, China, Lot: J11071	-
Anti-β-actin Antibody	Invitrogen, USA, Lot: RI2265993	1:5000
Anti-E-cadherin Antibody	Cell Signaling Technology, USA. Lot:6	1:1000
Anti-N-cadherin Antibody	Cell Signaling Technology, USA. Lot:12	1:1000
Anti-MMP2 Antibody	Cell Signaling Technology, USA. Lot:3	1:1000
Anti-MMP9 Antibody	Cell Signaling Technology, USA. Lot:5	1:1000
Anti-Vimentin Antibody	Proteintech, China, Lot: 00017056,	1:1000
Anti-Snail Antibody	Invitrogen, USA, Lot: PJ1921801	1:1000

Anti-Slug Antibody	Invitrogen, USA, Lot: QI2093091	1:1000
Anti-FN1 Antibody	Invitrogen, USA, Lot:3002128	1:1000
Anti-ITGA5 Antibody	Invitrogen, USA, Lot: UB2723355B	1:1000
0.2% TritonX-100	Proteintech, China, Lot: 61202011	-
TRIzol Reagent Solution	Ambion, Germany, Lot: 198912	-
Nuclei were stained using 4',6-diamidino-2-phenylindole (DAPI)	Beyotime Biotechnology, China, Lot: 091620210520	-
Horseradish peroxidase-conjugated goat anti-rabbit immunoglobulin (IgG)	Zhongshan Golden Bridge Biotechnology, China, Lot: 205001014	1:5000
Horseradish peroxidase-conjugated goat anti-mouse IgG	Zhongshan Golden Bridge Biotechnology, China, Lot: 203700821	1:5000
Horseradish peroxidase-conjugated rabbit anti-goat IgG	Zhongshan Golden Bridge Biotechnology, China, Lot: 20500927	1:5000
F-actin Antibody for Immunofluorescence	Servicebio, China, Lot: 1591610110103	1:500
phorbol 12-myristate 13-acetate (PMA)	Sigma-Aldrich, USA, Lot: SLBX8899	10 ng/mL
anti-CD163 polyclonal antibodies	Proteintech, China, Lot: 00091171	1:1000
anti-CD206 polyclonal antibodies	Proteintech, China, Lot: 00089604	1:1000
Alexa Fluor 488 AffiniPure goat anti-mouse IgG (H+L)	FcMACS, China, Lot: 136908	1:2000
Goat anti-mouse IgG (H+L) CoraLite594	Proteintech, China, Lot: 20000154	1:1000
Goat anti-rabbit IgG (H+L) R-PE conjugate	Proteintech, China, Lot: 20000129	1:1000

3 Methods

3.1 Immunohistochemical staining (IHC)

The slides were incubated with the FSTL3 antibody after blocking the tissue sections with the protein blocking solution. The IHC results (intensity and extent of staining) were independently scored by two observers. The intensity of staining was graded as follows: 0, negative staining; 1, weak staining;

2, moderate staining; and 3, strong staining. The extent of staining was scored on the basis of the proportion of the positively-stained cells per specimen, as follows: 0, no positively stained cells; 1 <10% positively -stained cells; 2, 10–50% positively -stained cells; and 3, >50% positively-stained cells. The histochemistry score (H-SCORE), representing the proportion of the positively-stained cells and the intensity of expression, was calculated as follows: H-SCORE= $\sum(PI \times I)$ = (percentage of cells with weak intensity × 1) + (percentage of cells with moderate intensity × 2) + (percentage of cells with strong intensity × 3). In the formula, the PI represents the percentage of positive cells to the total number of cells in a particular field and I represents the intensity of staining. The H-SCORE ranges from 0 to 300, with a higher score representing stronger positive staining.

3.2 Western blot assessment

The proteins were extracted using a RIPA lysis buffer and was quantitatively detected using the Bradford method (Schleicher and Wieland, 1978). The samples containing the same amount of 20 µg proteins were separated by the 10% or 8% SDS-PAGE. Then, the proteins were transferred onto the PVDF membranes. After blocking with BSA, the membranes were incubated with specific primary antibodies at 4˚C overnight. The following day, the membranes were rinsed and incubated with the corresponding secondary antibodies. Finally, an ECL detection kit was employed to assess the densities. The β-actin protein was used as a reference.

3.3 Enzyme-linked immunosorbent assay (ELISA)

We used the ELISA kit to detect its protein content in the cell supernatant and the serum of the patients. The supernatant from the culture was collected in sterile tubes and the supernatant was carefully collected after centrifugation for 20 min at 2000 rpm.

After isolation from the blood samples through centrifugation (2500 rpm for 15 min), the serum specimens were stored at -80°C at once.

The ELISA was used to quantify the FSTL3/FN1/α5β1 concentrations in the cell supernatant or serum.

In brief, the analyses were performed in 96-well polyvinyl chloride (PVC) microtiter plates. The plates were coated with a conditioned medium at 4 °C overnight and then washed three times (PBS-Tween solution). The non-specific sites were blocked using the bovine serum albumin at 37 °C for 2 h. After rinsing once the antibody specific for FSTL3/FN-1/α5β1 was added to the plates at 4 °C overnight and the plates were washed followed by the incubation in the anti-rabbit secondary antibody conjugated to horseradish peroxidase (HRP) for 2 h at room temperature (26-28 °C). After another wash, the final reaction was determined by adding 100 µL of freshly prepared peroxidase substrate solution. The enzyme was blocked with 100 µl H_2SO_4·2 N. The concentrations of FSTL3/FN-1/α5β1 were determined according to the standard curve from the synthesized peptide, and results were represented in ng/mL. The optical density of each well was examined immediately using a microplate reader (Bio-tek synergy HT) at 450 nm.

3.4 Migration and invasion assays

To investigate the cell migration *in vitro*, the CRC cells with NC, sh-FSTL3, and oe-FSTL3 constructs were collected. Approximately 1×10^4 cells were incubated in the upper chamber containing 200 µL of serum-free culture media, while 300 µL of the media supplemented with 10%

bovine serum were placed in the lower chamber. After 24 h of incubation, the cells that failed to attach to the top chamber were scraped off, while cells that adhered to the lower chamber were fixed with 95% alcohol and stained with 0.05% crystal violet. Then, images of five random fields of the stained filters were captured under a microscope (×200). Invasion assays were performed under the same conditions as the Transwell migration assays, although the cells were placed in the 8 μm pore size Matrigel-coated invasion chamber inserts. Then, the cells that had traversed through the membrane and entered the lower chamber were counted, and images were taken with a light microscope at a magnification of 200×. The results represented the average of three independent experiments.

3.5 Wound healing assay

A wound-healing assay was performed using the HT-29/GFP and RKO/GFP cells to determine the migration potential of the cells. The CRC cells were plated onto the 6-well plates at a density of 4×10^5 cells per well and were allowed to grow for a 24 h period in serum-free media to reach a confluent monolayer. The serum-free media was removed and the confluent cell sheet was wounded by scratching the culture well surface with a 10 μL pipette tip. The fluorescence microscopic imaging was used to recorded at 12, 24, and 48 h with an inverted fluorescence microscope (Olympus CKX-41, Japan) (×200 magnification).

3.6 Colony formation assays

For the colony formation assay, 500 cells were seeded into a 6-well plate and cultured for about 14 days, and then the colonies (>5 cells per colony) were stained with 0.5% crystal violet at room temperature for 10 min and counted using a stereomicroscope.

3.7 Immunofluorescence staining

Briefly, the cells seeded on coverslips were fixed in ice-cold methanol for 10 min followed by permeabilization with 0.1% Triton X-100 for 10 min at room temperature. The cells were then blocked with 10% bovine serum albumin in the phosphate-buffered saline, 0.1% Tween followed by an overnight incubation (4 °C) with the primary antibody. This procedure was followed by incubation with the corresponding secondary antibodies. The nuclei were stained using 4′,6-diamidino-2-phenylindole (DAPI) for 3 min, and incubated in the dark for 3 min. The slides were washed with PBS four times, for 5 min each. Then, the slides were sealed with a sealing solution containing a fluorescence quencher, followed by observing and imaging under a fluorescence microscope.

3.7.1 Concentration screening with polybrene

Polybrene is a positively charged small molecule binding to the anions on the cell surface, improving the efficiency of the lentiviral transfection in the cells. The addition of polybrene can improve the efficacy of the transfection by 2- to 10-fold. Since different cells have different sensitivities to polybrene, a range of 1–10 μg/mL was used for screening at the beginning of the experiment. Finally, we settled on a working concentration of 2 μg/mL.s

3.7.2 Puromycin screening of the stably transfected cell lines

(1) The cell density in the 24-well plates was 5×10^4 cells/well.

(2) The screening medium, i.e., fresh medium containing different concentrations of puromycin (0–10 μg/mL), was prepared.

(3) The cells were incubated overnight in a screening medium.

(4) The old screening medium was replaced with a fresh screening medium every ~2–3 days.

(5) The surviving cells were observed daily, as the optimal duration of puromycin action generally ranges from 1 to 4 days.

(6) The minimum concentration of puromycin used was the lowest concentration that kills all cells within 1–4 days from the start of the screening.

(7) After screening, a concentration of 1.5 μg/mL was chosen.

3.7.3 Phorbol-12-myristate 13-acetate (PMA) induction scheme selection

The THP-1 cell line is a human leukemia cell-derived monocyte line capable of acquiring phenotypic and functional characteristics similar to those of the primary the macrophages upon PMA stimulation (Tsuchiya et al., 1982). It is currently the most widely used *in vitro* model of human-derived macrophages. It has been proven that 5 ng/mL PMA treatment can cause THP-1 monocytes to differentiate into macrophages (Genin et al., 2015). In addition, it has been presumed by many researchers that the higher the PMA concentration makes it easier for the differentiated macrophages to transform into the M1 subtype (Chanput et al., 2013). Therefore, a low-concentration induction protocol was chosen for this study. Due to various inter-laboratory variations, the initial induction with 5 ng/mL PMA for 48 h resulted in the adherence of only about 60% of the THP-1 cells to the wall. Since 95% of THP-1 cells differentiated and adhered to the wall under this treatment condition, subsequently, after many adjustments, 10 ng/mL for 48 h was selected as the induction model.

3.7.4 Concentration screening for ATN-161

ATN-161: A small peptide antagonist of integrin α5β1.

The cell viability was determined using a CCK-8 kit. Briefly, the cells were harvested and plated at a density of 4,000 cells/well in a 200 µL fresh growth medium containing 10% FBS in 96-well plates. Subsequently, the cells were treated with different concentrations of LY364947 for 24 h at 37 °C.

At the indicated time-points, the CCK-8 (10 µL per 100 µL medium) was added to each well, and the cells were incubated for 2 h at 37 °C.

The optical density was measured at 450 nm using a multimode microplate reader (BioTek Synergy HT).

Each assay was performed in triplicate. Finally, the cell growth inhibitory rates were determined using the calibration curves.

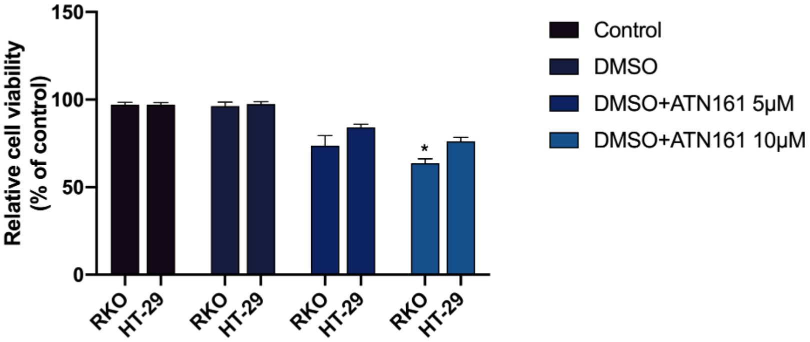

Reference

Chanput, W., Mes, J.J., Savelkoul, H.F., and Wichers, H.J. (2013). Characterization of polarized THP-1 macrophages and polarizing ability of LPS and food compounds. *Food Funct.* 4**,** 266-276.

Genin, M., Clement, F., Fattaccioli, A., Raes, M., and Michiels, C. (2015). M1 and M2 macrophages derived from THP-1 cells differentially modulate the response of cancer cells to etoposide. *BMC Cancer* 15**,** 577.

Schleicher, E., and Wieland, O.H. (1978). Evaluation of the Bradford method for protein determination in body fluids. *J. Clin. Chem. Clin. Biochem.* 16**,** 533-534.

Tsuchiya, S., Kobayashi, Y., Goto, Y., Okumura, H., Nakae, S., Konno, T., and Tada, K. (1982). Induction of maturation in cultured human monocytic leukemia cells by a phorbol diester. *Cancer Res.* 42**,** 1530-1536.
